# Progressive cholinergic decline in Alzheimer's Disease: consideration for treatment with donepezil 23 mg in patients with moderate to severe symptomatology

**DOI:** 10.1186/1471-2377-11-21

**Published:** 2011-02-07

**Authors:** Marwan Sabbagh, Jeffrey Cummings

**Affiliations:** 1 The Cleo Roberts Center for Clinical Research, Banner Sun Health Research Institute, Sun City, Arizona, USA; 2 The Cleveland Clinic Lou Ruvo Center for Brain Health, Las Vegas, Nevada, and Cleveland, Ohio, USA

## Abstract

Of the estimated 5.3 million people with Alzheimer's disease in the United States, more than half would be classified as having moderate or severe disease. Alzheimer's disease is a progressive disorder with the moderate to severe stages generally characterized by significant cognitive, functional, and behavioral dysfunction. Unsurprisingly, these advanced stages are often the most challenging for both patients and their caregivers/families. Symptomatic treatments for moderate to severe Alzheimer's disease are approved in the United States and include the acetylcholinesterase inhibitor donepezil and the glutamate receptor antagonist memantine. Progressive symptomatic decline is nevertheless inevitable even with the available therapies, and therefore additional treatment options are urgently needed for this segment of the Alzheimer's disease population. An immediate-release formulation of donepezil has been available at an approved dose of 5-10 mg/d for the past decade. Recently, the United States Food and Drug Administration approved a higher-dose (23 mg/d) donepezil formulation, which provides more gradual systemic absorption, a longer time to maximum concentration (8 hours) versus the immediate-release formulation (3 hours), and higher daily concentrations. Herein, we review (1) the scientific data on the importance of cholinergic deficits in Alzheimer's disease treatment strategies, (2) the rationale for the use of higher-dose acetylcholinesterase inhibitors in patients with advanced disease, and (3) recent clinical evidence supporting the use of higher-dose donepezil in patients with moderate to severe Alzheimer's disease.

## Background

Alzheimer's disease (AD) is a progressive disorder that advances from a period of mild cognitive impairment through moderate and severe stages. There are currently an estimated 5.3 million people with AD in the United States [1], and more than half of these individuals will likely be categorized as having moderate or severe disease [2]. These advanced stages of AD extend over a period of several years and are often the most difficult for both patients and caregivers [1].

As AD progresses toward the more advanced stages, symptoms worsen, and the need for effective treatments grows more critical; yet, clinicians have few options. Only 2 agents are currently approved in the United Stated for treatment beyond the mild to moderate stage--donepezil, an acetylcholinesterase inhibitor (AChEI), and memantine, a glutamate receptor antagonist that can be used alone or in combination with an AChEI [3,4]. Progression of AD is inevitable, however, and cannot be halted by these therapy options, which address primarily the disease symptoms. Given the limited choices for therapy and the often devastating and prolonged impact of further decline in cognitive and functional abilities, additional options for treatment are urgently needed for this segment of the AD population.

In the development of AChEIs, determination of appropriate dosing was typically based on decisions regarding a balance of efficacy versus tolerability. For the past decade, the immediate-release formulation of donepezil has been used at an approved dose of 5-10 mg/d [3]. However, a higher-dose (23 mg/d) donepezil formulation was recently approved in the United States that provides more gradual systemic absorption, a longer time to maximum concentration (8 hours) compared with the immediate-release formulation (3 hours), and higher concentrations. In this article we provide information on the effects of donepezil on the cholinergic system and review the scientific and clinical evidence supporting the use of higher-dose donepezil in patients with moderate to severe AD.

## Importance of cholinergic deficit in the treatment of AD

An important component of the pathophysiology of AD, recognized more than 30 years ago, is degeneration of the cholinergic system [5]. Early histologic studies showing loss of cholinergic activity as AD progresses are supported by several modern lines of investigation using advanced imaging techniques, including positron emission tomography (PET) and magnetic resonance imaging (MRI). PET imaging has consistently demonstrated reductions in cortical AChE activity in patients with mild or moderate AD compared with normal controls [6-8], and these deficits have been correlated with increasing cognitive decline [9]. In addition, atrophy of the nucleus basalis of Meynert, the major source of the cholinergic neurotransmitter acetylcholine (ACh), its primary synthesizing enzyme choline acetyltransferase, and its primary metabolizing enzyme AChE, is notable in patients with AD on MRI [10,11].

Appreciable loss of cortically projecting cholinergic neurons occurs as a hallmark of the disease, particularly in brain areas associated with memory and learning (ie, the hippocampus, nucleus basalis of Meynert, and cortex) [12,13]. Reduction of cholinergic activity in AD patients has been observed to correlate with cognitive deficits as measured by dementia rating scales [14].

The cholinergic abnormalities seen in AD are not viewed as the cause of the disorder, but cholinergic involvement is significant because it is universal, correlates with cognitive defects, and is one of the few pathophysiologic phenomena that can be addressed with currently approved treatment options. Enhancement of cognitive function occurs when the action of ACh is increased via inhibition of its metabolizing enzymes, principally AChE [13]. Accordingly, the strategy of increasing cholinergic activity to mitigate the cognitive symptoms of AD has been a primary and enduring therapeutic tactic.

## Rationale for using higher-dose AChEIs in patients with moderate to severe AD

### Levels of AChE inhibition may be suboptimal

Inhibition of AChE in peripheral red blood cells (RBCs) has been used as a convenient surrogate marker for the activity of centrally acting AChEIs [15]. With the use of RBC assays, the level of AChE inhibition at the highest approved doses of donepezil, rivastigmine, and galantamine has been reported to be approximately 50% to 80% [16]. These findings led to the suggestion that current dosing options provide adequate AChE inhibition.

Functional imaging studies, however, suggest that RBC-based assays may overestimate AChE inhibition in the brain [8]. PET imaging studies have shown that stable regimens of donepezil 5 mg/d or 10 mg/d, or rivastigmine 9 mg/d, inhibit in vivo cortical AChE activity by approximately 20% to 40% in patients with mild to moderate AD [8,17]. The finding that current dosing schedules may not achieve the maximum inhibition of peripheral or central nervous system AChE activity suggests that higher doses of AChEIs may facilitate greater AChE inhibition and that this may subsequently increase efficacy [8].

### Cholinergic dysfunction is more pronounced in advanced stages of AD

Data from studies that examined brain tissue samples obtained during biopsy or autopsy indicate that the cholinergic deficit is most evident in patients with more advanced symptoms [14]. This suggests that the target level of cholinergic enhancement may change as symptoms progress. Patients in early stages of AD may achieve sufficient cholinergic stimulation from lower doses of AChEIs, while higher AChEI doses may be required in patients with more advanced AD.

## Utility of higher doses of AChEIs: 5 mg/d versus 10 mg/d donepezil

Clinical trials in which patients with AD were randomized to treatment with donepezil 5 mg/d or 10 mg/d or placebo have demonstrated a relationship between benefits in cognition and higher donepezil dose [18,19]. Notably, the benefits of a higher dose of donepezil were most apparent in trials including patients with more advanced AD [20].

Results from 3 similarly designed placebo-controlled clinical trials that assessed the safety and efficacy of donepezil 5 mg/d and 10 mg/d in patients with mild to moderate AD (baseline Mini-Mental State Examination [MMSE] score of 10-26) were published in the period after the initial US Food and Drug Administration (FDA) approval of donepezil in 1996. In each trial, co-primary outcomes were the Alzheimer's Disease Assessment Scale-cognitive subscale (ADAS-cog) and the Clinician's Interview-Based Impression of Change-plus caregiver input (CIBIC-plus, a measure of global function) [21-23]. Subjects were treated for either 12 weeks [22] or 24 weeks [21,23].

In all 3 studies, significant benefits compared with placebo were seen in both co-primary variables at study end point with donepezil 5 mg and 10 mg. The magnitude of benefit was numerically greater with donepezil 10 mg compared with 5 mg across the 3 studies, but either between-dose differences were not statistically significant [22] or statistical comparisons were not reported [21,23]. More recently, however, a meta-analysis of 10 randomized, double-blind, placebo-controlled donepezil studies involving 2376 patients, which included the aforementioned 3 studies, found that improvements in ADAS-cog at 18 and 24 weeks were significantly greater with donepezil 10 mg compared with donepezil 5 mg (p = 0.015 and p = 0.005, respectively) [19]. The meta-analysis also reported an increase in cholinergic adverse events (AEs) when the dose was increased from 5 mg/d to 10 mg/d. However, this increase was generally attributed to the outdated practice of increasing the donepezil dose after just 1 week of treatment, and the authors concluded that both doses were well tolerated in this study population.

Results from a recent study published by Nozawa et al [20] suggest that increasing the dose of donepezil may be beneficial to patients with more advanced AD symptoms. In this open-label study, patients categorized with mild, moderate, or advanced AD (based on a Clinical Dementia Rating scale score of 1, 2, and 3, respectively) who had been on a stable regimen of donepezil 5 mg/d were increased to donepezil 10 mg/d. Patients with mild or moderate AD demonstrated no significant decline in MMSE or Revised Hasegawa Dementia Scale (HDS-R) scores during the 6 months before or after donepezil was increased from 5 mg/d to 10 mg/d. However, in the more advanced AD group, which had a mean baseline MMSE of 12.7, MMSE and HDS-R scores had decreased significantly during the 6 months prior to dose increase but no additional declines were observed in the 6 months following dose increase. During this study, 7 of 61 patients (11.5%) withdrew due to AEs that emerged after their dose was increased from 5 mg/d to 10 mg/d; however, all AEs appeared in these patients within 1 month of the dose increase and symptoms rapidly improved after dose modification back to 5 mg/d [20].

Outcomes from a donepezil dose-ranging clinical trial also suggest that higher doses of donepezil are more effective and similarly well tolerated in patients with advanced AD [18]. In this blinded study, patients with a baseline MMSE score of 1 to 12 were treated for 24 weeks with donepezil 10 mg/d, donepezil 5 mg/d, or placebo. Among the study population, mean baseline scores on the Severe Impairment Battery (SIB), a scale designed specifically to assess cognition in patients with more severe cognitive dysfunction, ranged from 56.7 to 67.0 across the 3 treatment groups. Co-primary end points were CIBIC-plus and SIB. At study end point, mean SIB scores with donepezil 10 mg/d and donepezil 5 mg/d had improved (+4.7 points and +2.5 points, respectively), whereas the mean score in the placebo group had declined by 4.2 points; differences between mean SIB scores for both donepezil doses and for the placebo group were statistically significant. On the CIBIC-plus, donepezil 10 mg/d resulted in significant benefits over placebo, but no significant benefits over placebo were seen with the 5 mg/d dose. Notably, a significant dose-response relationship was demonstrated on both the SIB and the CIBIC-plus (p ≤ 0.003), suggesting that patients with advanced AD are most likely to receive benefit from higher levels of AChE inhibition. As seen in the mild to moderate AD population, an increased incidence of treatment-emergent AEs (TEAEs) was observed in the donepezil 10 mg/d group (83.3%) versus the donepezil 5 mg/d group (78.2%) in this study. However, this difference was not statistically significant and the higher dose was seen to be safe and well tolerated in patients with advanced AD [18].

## Investigation of doses of donepezil in excess of 10 mg/d

Data showing improved results with 10 mg/d versus 5 mg/d donepezil, along with findings suggesting that patients in more advanced stages of AD show a more pronounced effect with higher dosing, led to trials testing doses higher than 10 mg/d [24,25]. In a 24-week pilot study (N = 31) designed to assess the safety and tolerability of higher than standard doses of donepezil in patients with mild to moderate AD (MMSE score, 10-26) on a stable 10 mg/d donepezil regimen, once-daily administration of up to 20 mg was found to be safe and well tolerated. No differences in efficacy outcomes were reported; this study was not powered to show statistical differences between groups on clinical outcomes [24].

To test the hypothesis that patients with more advanced AD can benefit from an increased dose of established AChEI therapy, a study was designed to evaluate the safety and efficacy of a donepezil 23 mg tablet. This new donepezil formulation was developed to provide a higher dose administered once daily and to avoid sharp increases in peak concentration.

**Figure 1:**
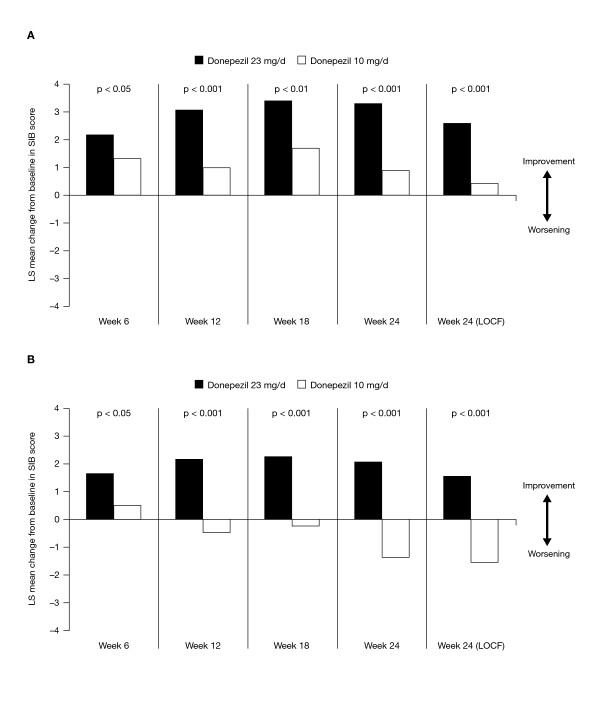
**LS mean SIB total score change from baseline by Visit (observed cases) and at primary end point (Week 24-LOCF) in patients with MMSE scores of 0-20 (primary analysis; A) and 0-16 (post hoc analysis; B)**.

Results of this phase 3 trial to assess the safety and efficacy of the 23 mg/d donepezil tablet have recently been published [25], and this treatment has been subsequently approved by the FDA for use in patients with moderate to severe AD. In this 24-week randomized, double-blind, global, head-to-head clinical trial, more than 1400 patients with moderate to severe AD (MMSE, 0-20) on a stable donepezil 10 mg/d regimen for ≥ 3 months were randomized to increase their dosages to the once-daily donepezil 23 mg tablet or to be maintained on the 10 mg/d dose. The co-primary end points were changes in SIB score and the CIBIC-plus overall change score at Week 24. Patients in the donepezil 23 mg group showed a statistically significant improvement in cognition as measured by the SIB compared with donepezil 10 mg (Figure 1A). The least squares (LS) mean difference between treatments for the change from baseline to Week 24 for the 23 mg and 10 mg groups was 2.2 points (p < 0.001). Among the total study population (MMSE, 0-20), significant incremental benefit on the CIBIC-plus was not seen with donepezil 23 mg/d versus donepezil 10 mg/d over 24 weeks of treatment.

In a post hoc analysis of patients with more severe cognitive impairment (baseline MMSE, 0-16), significant differences favoring donepezil 23 mg/d were demonstrated on both the SIB (Figure 1B) and the CIBIC-plus. In this subpopulation, representing 70% of patients in the study, the LS mean between-treatment difference in change in SIB score was 3.1 points (p < 0.0001) at Week 24. CIBIC-plus scores at Week 24 were 4.31 and 4.42 in the 23 mg and 10 mg groups, respectively (p = 0.0279).

During the study, TEAEs were reported in more patients receiving donepezil 23 mg/d (73.7%) than receiving donepezil 10 mg/d (63.7%) [25]. The most common TEAEs considered probably related to treatment with donepezil 23 mg/d and 10 mg/d were nausea (6.1% vs 1.9%), vomiting (5.0% vs 0.8%), and diarrhea (3.2% vs 1.5%). In the 23 mg/d group, gastrointestinal AEs occurred most frequently during the first month of treatment, but were relatively infrequent beyond this time. Serious TEAEs were noted in a similar proportion of patients in the 23 mg/d and 10 mg/d groups (8.3% vs 9.6%), with the majority in both groups considered unrelated to treatment. Overall, 18.6% of patients treated with donepezil 23 mg discontinued due to TEAEs, compared with 7.9% remaining on donepezil 10 mg; the majority (>60%) of discontinuations from the 23 mg group occurred in the first month of therapy.

This 24-week study demonstrated that treatment with donepezil 23 mg tablets can provide additional cognitive benefit in patients with moderate to severe AD compared with 10 mg donepezil, and its findings are consistent with previous analyses showing greater benefit with 10 mg than with 5 mg [18-20]. Although TEAEs were more commonly observed upon initiation of the 23 mg/d dose, as was expected based on prior experience of dose increases from 5 mg/d to 10 mg/d, the higher dose was generally well tolerated, with a safety profile typical of AChEIs. Moreover, strategies required to manage AEs emerging upon a dose increase to 23 mg/d should be familiar to health care providers already treating patients with standard donepezil regimens. Overall, although further investigations are needed to determine the long-term benefits provided by donepezil 23 mg, the results described by Farlow and colleagues [25] offer evidence that the use of higher doses of AChEIs may be a worthwhile goal to improve treatment of AD in patients with more advanced disease.

## Conclusions

Evidence indicates that cholinergic deficits correlate with the cognitive defects of AD. As AD symptoms increase in severity over time, there are progressive declines in markers of cholinergic activity. Inhibition of AChE increases cholinergic function, which is theorized to result in dose-dependent improvements in cognition.

Patients with AD continue to decline even when treated with the highest currently approved doses of AChEIs, and patients with advanced disease demonstrate the most profound impairment in cholinergic function. Imaging studies have demonstrated that, with the currently used doses of AChEIs, inhibition of brain AChE is suboptimal. Data from a recently published 24-week clinical trial in patients with moderate to severe AD demonstrate that treatment with a 23 mg formulation of donepezil results in significant benefits in cognition compared with standard doses, with an acceptable tolerability profile that is consistent with the AChEI class. Donepezil 23 mg did not differ from the 10 mg dose in terms of the incidence of serious side effects and differences in discontinuation rates between the two doses were not evident after the first month of treatment. Once-daily donepezil 23 mg, therefore, represents an additional treatment choice for patients with advanced AD.

## Competing interests

**MS: **Dr Sabbagh has worked in an advisory/consulting capacity for Eisai, Pfizer, Amerisciences, Takeda and GSK; has received royalties from Wiley and Amerisciences; and has grants and contracts with Lilly, Baxter, Bayer, GE, Bristol-Myers Squibb, Eisai, Janssen, Wyeth/Elan, Avid, and Medivation.

**JC: **Dr Cummings has provided consultation to Abbott, Acadia, Acerra, ADAMAS, Astellas, Avanir, Bristol-Myers Squibb, CoMentis, Eisai, Elan, EnVivo, Forest, Genentech, GlaxoSmithKline, Janssen, Lilly, Lundbeck, Medivation, Medtronics, Merck, Merz, Myriad, Neurokos, Novartis, Orion, Pfizer, Prana, reMYND, Signum Bioscience, Sonexa, Takeda, and Toyama pharmaceutical companies. Dr Cummings has also served as a speaker/lecturer for Eisai, Forest, Janssen, Novartis, Pfizer, Lundbeck, Merz

## Authors' contributions

**MS: **Dr Sabbagh was involved in the drafting of the manuscript and revised it for important intellectual content. **JC: **Dr Cummings was involved in the drafting of the manuscript and revised it for important intellectual content. **MS + JC: **Dr Sabbagh and Dr Cummings reviewed and fully approved the final version of the manuscript for submission.

## Pre-publication history

The pre-publication history for this paper can be accessed here:

http://www.biomedcentral.com/1471-2377/11/21/prepub
